# Distributed Optimization for Resource Allocation Problem with Dynamic Event-Triggered Strategy

**DOI:** 10.3390/e25071019

**Published:** 2023-07-04

**Authors:** Feilong Guo, Xinrui Chen, Mengyao Yue, Haijun Jiang, Siyu Chen

**Affiliations:** College of Mathematics and System Sciences, Xinjiang University, Urumqi 830017, China; feilongguo2022@163.com (F.G.); xinrui_chen0925@163.com (X.C.); 15276694756@163.com (M.Y.); sychenyy@163.com (S.C.)

**Keywords:** resource allocation problem, distributed optimization, dynamic event-triggered, consensus, Zeno behavior

## Abstract

This study aims to unravel the resource allocation problem (RAP) by using a consensus-based distributed optimization algorithm under dynamic event-triggered (DET) strategies. Firstly, based on the multi-agent consensus approach, a novel one-to-all DET strategy is presented to solve the RAP. Secondly, the proposed one-to-all DET strategy is extended to a one-to-one DET strategy, where each agent transmits its state asynchronously to its neighbors. Furthermore, it is proven that the proposed two types of DET strategies do not have Zeno behavior. Finally, numerical simulations are provided to validate and illustrate the effectiveness of the theoretical results.

## 1. Introduction

With the development of network information technology and the era of artificial intelligence, multi-agent systems (MASs) have received extensive attention in view of their applications in the machining industry [1], synchronous generators [2], microservice-based cloud applications [3], USVs [4], and other fields. It is worth noting that consensus is one of the most fundamental and important problems in MASs, and there have been many studies about it [5,6,7,8]. In essence, the distributed optimization problem is that a group of agents achieve a goal by exchanging local information with neighbors and minimizing the sum of all the local cost functions. In contrast to conventional consensus, distributed optimization problems require both achieving consensus and solving optimization problems. Up to now, distributed optimization problems have already appeared widely in power systems [9], MPC and network flows [10], wireless ad hoc networks [11], etc.

Early distributed optimization problems were mainly solved by centralized optimization algorithms. The feature of a centralized optimization algorithm is that all agents have a central node that centrally stores all of the information to address the optimization problem [12,13]. However, centralized optimization algorithms are unsuitable for large-scale networks, because collecting information from all agents in the network requires a lot of communication and computational overhead, and there will be the single point of failure problem. Consequently, distributed optimization algorithms have emerged as the times require. In recent years, distributed optimization algorithms are divided into two main categories, i.e., discrete-time algorithms and continuous-time algorithms. More specifically, discrete-time distributed optimization algorithms have been utilized in the optimal solution of the saddle point dynamics problem [14], epidemic control resource allocation [15], and tactical production planning [16]. Additionally, many researchers have made substantial explorations of continuous-time distributed optimization algorithms recently. For instance, a continuous-time optimization model was developed in [17] for source-sink matching in carbon capture and storage systems. In [18], the application of a continuous-time optimization algorithm was investigated in power system load distribution, and the distributed continuous-time approximate projection protocol was proposed in [19] for solving the shortest distance optimization problem.

Many of the above optimization algorithms communicate in continuous time, which can lead to frequent algorithm updating and then cause unnecessary communication resource consumption, so it is necessary to solve the system’s resource problem. Therefore, applying event-triggered strategies to distributed optimization algorithms [20,21,22,23,24,25,26] is a feasible and promising scheme that can effectively reduce the energy waste of the system. Only when the designed event-triggered condition is satisfied, is the system allowed to communicate and update the protocol, which helps to reduce the cost and burden of communication and computing as well as the collection of gradient information. Primarily, for static event-triggered (SET) mechanisms, which include the constant trigger thresholds independent of time, it is theoretically difficult to rule out Zeno behavior. Furthermore, as the working time increases, the inter-event time intervals become larger, which results in more trigger actions and wasting the system’s resources. Furthermore, the event-triggered strategy has undergone a paradigm shift from the SET strategy to the dynamic event-triggered (DET), which introduces an auxiliary parameter for each agent to dynamically adjust its threshold. Moreover, in most cases, the DET strategy can well extend the average event intervals, thus further reducing the consumption of communication resources compared to SET communication. Therefore, the DET strategy has aroused much interest and it holds great applicability value, which was considered in [27,28,29,30,31,32,33]. An improved event-triggered strategy, independent of the initial conditions, was leveraged in [34] to solve the topology separation problems caused by critical communication link failures. In [35], the corresponding DET mechanism was presented for two cases based on nonlinear relative and absolute states coupling, and it was also proved that the continuous communication between agents can be effectively avoided. Under the DET strategy, each agent transmits information to all neighbors synchronously when its trigger condition is met, which is usually called the one-to-all DET strategy. Nevertheless, under the one-to-all DET strategy, it is unreasonable to ignore the possibility that each agent has different triggering sequences. Therefore, to overcome the limitation of the one-to-all DET strategy, it is essential to design a DET strategy that allows each agent to decide its own triggering sequences and transmit information asynchronously to its neighbors according to different event-triggered conditions designed for each of its neighbors, which is referred to as the one-to-one DET strategy. Under the one-to-one DET strategy, owing to its characteristics, an agent is not constrained by any synchronous execution of its neighbors’ transmission information, so it can adjust the information transmission more flexibly, especially in the case of cyber-attacks. In [36], under an adaptive DET strategy, the fully distributed observer-based strategy was developed, which guarantees asymptotic consensus and eliminates Zeno behavior.

So far, note that many distributed optimization algorithms have been leveraged to solve the resource allocation problem (RAP), such as in [37,38,39]. Therefore, it is necessary and significant to combine DET strategies to solve the RAP. Motivated by the above discussions, we further investigate distributed optimization algorithms with two novel synchronous and asynchronous DET strategies to address the RAP. The main contributions of this article are developed as follows.

(1) This work combines the consensus idea and one-to-all DET strategy to design a new distributed optimization algorithm to solve RAP, in which the algorithm can keep the equality constraint constant. In addition, unlike the SET strategies of [40,41], the DET in this work has a lower trigger frequency, which means that the system resources can be saved.
(2) In order to improve the flexibility and practicality of the algorithm, the one-to-all DET strategy is extended to a one-to-one DET strategy. Based on this strategy, a distributed optimization algorithm is developed to address the RAP.
(3) The two types of proposed distributed optimization algorithms only use the information of the decision variable $x_{i}\left(t\right)$ to avoid the communication among agents, which ingeniously reduces the resource consumption, while the algorithm in [42] needs to exchange information about the variables $\phi_{i}\left(t\right)$ and $\zeta_{i}\left(t\right)$. In addition, the introduced internal dynamic parameters in this work are not only effective in solving RAP, but also crucial in successfully excluding Zeno behavior.

The organization of the remaining parts of this paper is as follows. Some algebraic graph theory preliminaries, a basic definition and assumptions, and the optimization problem formulation are given in Section 2. In Section 3 and Section 4, distributed optimization algorithms under the proposed one-to-all and one-to-one DET strategies are presented to solve the RAP. Furthermore, the proof of the exclusion of Zeno behavior is included. In Section 5, numerical simulation results are given to illustrate the effectiveness of the proposed algorithms. Finally, we show our conclusions and future work direction in Section 6.

**Notation** **1.** *The symbols appearing in this article are listed in Table 1.*

## 2. Preliminaries

### 2.1. Algebraic Graph Theory

The topology among *n* nodes can be modeled as a graph G=(V,E,A) consisting of a finite node set $V=(v_{1},v_{2},\cdots ,v_{n})$, a set of edges E⊆V×V, and a weighted adjacency matrix $A=\left[a_{ij}\right]\in R^{n\times n}$, with $a_{ij}>0$ if $(v_{j},v_{i})\in E$ and $a_{ij}=0$ otherwise. Given an edge $(v_{j},v_{i})\in E$, we refer to $v_{j}$ as a neighbor of $v_{i}$, then, $v_{j}$ and $v_{i}$ can receive each other’s information. The set of $v_{i}$ is defined as $N_{i}=\left\{v_{j}\in V:\left(v_{i},v_{j}\right)\in E\right\}$, which does not contain self-edges $\left(v_{i},v_{i}\right)$. An undirected graph G is connected if for any vertex $v_{i},\;v_{j}\in V$, there exists a path that connects $v_{i}$ and $v_{j}$. The Laplacian matrix $L=\left[l_{ij}\right]\in R^{n\times n}$ is denoted by $l_{ii}=\sum_{j=1,j\neq i}^{n}a_{ij}$ and $l_{ij}=-a_{ij},\;i\neq j$. Furthermore, $1_{n}^{⊤}L=0_{n}^{⊤}$. The eigenvalue of L is a non-decreasing order, i.e., $0=\lambda_{1}<\lambda_{2}\leq \cdots \leq \lambda_{n}$.

### 2.2. Problem Statement

In the distributed RAP, we consider the MASs composed of *n* agents where each agent has a local quadratic convex cost function $f_{i}\left(x_{i}\left(t\right)\right):R\to R$. The global objective function is denoted by $F\left(x\left(t\right)\right):R^{n}\to R$. $f_{i}\left(x_{i}\left(t\right)\right)=\alpha_{i}x_{i}^{2}\left(t\right)+\beta_{i}x_{i}\left(t\right)+\gamma_{i}$, where the cost coefficients $\alpha_{i}$, $\beta_{i}$, and $\gamma_{i}>0$. Then, the RAP can be rewritten as the following optimization problem:

(1) $$\begin{array}{c} \left\{\begin{array}{cc} \min & F\left(x\left(t\right)\right)=\sum_{i=1}^{n}f_{i}\left(x_{i}\left(t\right)\right),\\ s.t. & \sum_{i=1}^{n}x_{i}\left(t\right)=D, \end{array}\right. \end{array}$$

where $x\left(t\right)={\left(x_{1}\left(t\right),x_{2}\left(t\right),\cdots ,x_{n}\left(t\right)\right)}^{⊤}$ is a decision variable vector. For convenience, only the case $x_{i}\left(t\right)\in R$ will be discussed, owing to the fact that when $x_{i}\left(t\right)\in R^{n}$ it can be solved similarly and completely by using the Kronecker product. F(x(t)) and $f_{i}\left(x_{i}\left(t\right)\right)$ stand for the global cost function and the local cost function, respectively. D∈R represents the global resource constraint. In the economic dispatch problem of smart grids, $x_{i}\left(t\right)$ denotes the output power of generator *i*, and *D* denotes the total power demand and equality constraint and is called the demand constraint.

This paper aims to design distributed DET strategies to solve RAP (1). Therefore, we need the following definition and assumptions before further analysis.

**Definition** **1.** *The multi-agent consensus problem would be addressed as long as for any initial value of state $z_{i}\left(0\right)\in R^{n}$,*

$$\lim_{t\to \infty }∥z_{i}\left(t\right)-z_{j}\left(t\right)∥=0,\;\forall i,j\in N.$$

**Assumption** **1.** *The communication topology is undirected and connected.*

**Assumption** **2.** *The local objective functions are quadratically continuously differentiable and strongly convex.*

## 3. The One-to-All DET Strategy

In this section, we construct the one-to-all DET strategy, which allows each agent to transmit information synchronously. Moreover, a distributed optimization algorithm with the proposed DET is introduced and the consensus is derived, which solves the RAP (1).

For the one-to-all DET, the triggering time sequence is determined by $0=t_{0}^{i}<t_{1}^{i}<\cdots <t_{s}^{i}<\cdots .$ The measurement error of each agent is defined as

$$e_{i}\left(t\right)=\nabla f_{i}\left(x_{i}\left(t_{k}^{i}\right)\right)-\nabla f_{i}\left(x_{i}\left(t\right)\right),t\in \left[t_{k}^{i},t_{k+1}^{i}\right),\forall i\in N.$$

Then, we propose the one-to-all DET triggering sequence ${\left\{t_{k}^{i}\right\}}_{k\in N}$ as follows

(2a) $$t_{k+1}^{i}=\underset{l>t_{k}^{i}}{\inf}\left\{l:{\left(e_{i}\left(t\right)\right)}^{2}-c_{i}\sum_{j=1}^{n}a_{ij}{\left(\phi_{i}\left(t\right)-\phi_{j}\left(t\right)\right)}^{2}-\pi_{i}\Gamma_{i}\left(t\right)\geq 0,\forall t\in \left[t_{k}^{i},l\right]\right\},$$

where $c_{i}$ and $\pi_{i}$, ∀i∈N, are positive constants.

**Remark** **1.** *If setting $\Gamma_{i}\left(t\right)=0$, the DET strategy reduces to the SET strategy. Then, the one-to-all SET triggering sequence ${\left\{t_{k}^{i}\right\}}_{k\in N}$ is as follows*

(2b) $$\begin{array}{c} t_{k+1}^{i}=\underset{l>t_{k}^{i}}{\inf}\left\{l:{\left(e_{i}\left(t\right)\right)}^{2}-c_{i}\sum_{j=1}^{n}a_{ij}{\left(\phi_{i}\left(t\right)-\phi_{j}\left(t\right)\right)}^{2}\geq 0,\forall t\in \left[t_{k}^{i},l\right]\right\}. \end{array}$$

*Consequently, the SET strategy is a special case of the DET strategy, and the DET strategy is a more general situation. In addition, due to the internal dynamic variables of the DET function, it is easier to exclude Zeno behavior than for SET.*

Inspired by [43], we design an internal dynamic variable $\Gamma_{i}\left(t\right)$ satisfying

(3) $${\dot{\Gamma }}_{i}\left(t\right)=-\psi_{i}\Gamma_{i}\left(t\right)+\mu_{i}\left[c_{i}\sum_{j=1}^{n}a_{ij}{\left(\phi_{i}\left(t\right)-\phi_{j}\left(t\right)\right)}^{2}-{\left(e_{i}\left(t\right)\right)}^{2}\right],$$

where $\Gamma_{i}\left(0\right)>0$, $\psi_{i}$ and $\mu_{i}=\frac{w_{i}}{c_{i}}$ with $w_{i}\geq 0,$∀i∈N, are positive constants.

Let $\hat{\phi_{i}}\left(t\right)=\nabla f_{i}\left(x_{i}\left(t_{k}^{i}\right)\right)$ and $\phi_{i}\left(t\right)=\nabla f_{i}\left(x_{i}\left(t\right)\right)$. The distributed optimization algorithm is designed as follows to solve the RAP (1):

(4) $$\left\{\begin{array}{c} x_{i}\left(t\right)=\zeta_{i}\left(t\right)+x_{i}\left(0\right),\\ {\dot{\zeta }}_{i}\left(t\right)=\sum_{j=1}^{n}a_{ij}\left(\hat{\phi_{j}}\left(t\right)-\hat{\phi_{i}}\left(t\right)\right),\\ \zeta_{i}\left(0\right)=0, \end{array}\right.$$

where $\zeta_{i}\left(t\right)$, ∀i∈N is an auxiliary variable.

According to the distributed optimization algorithm (4), one obtains ${\dot{\phi }}_{i}\left(t\right)=\frac{\partial^{2}f_{i}\left(x_{i}\left(t\right)\right)}{\partial x_{i}^{2}\left(t\right)}$${\dot{x}}_{i}\left(t\right)=2\alpha_{i}{\dot{\zeta }}_{i}\left(t\right)=2\alpha_{i}\sum_{j=1}^{n}a_{ij}\left[\hat{\phi_{j}}\left(t\right)-\hat{\phi_{i}}\left(t\right)\right],$ where the initial value $\phi_{i}\left(0\right)$ satisfies $\phi_{i}\left(0\right)=2\alpha_{i}x_{i}\left(0\right)+\beta_{i}.$

In addition, ${\dot{\phi }}_{i}\left(t\right)$ in matrix form can be described as

$$\dot{\phi }\left(t\right)=-2\Lambda L\left[\phi \left(t\right)+e\left(t\right)\right],$$

where $\phi \left(t\right)={\left(\phi_{1}\left(t\right),\phi_{2}\left(t\right),\;\cdots ,\phi_{n}\left(t\right)\right)}^{⊤}\in R^{n}$, $\Lambda =\mathrm{diag}\{\alpha_{1},\cdots ,\alpha_{n}\}$ and $e\left(t\right)=(e_{1}\left(t\right),$$e_{2}\left(t\right),\cdots ,e_{n}{\left(t\right))}^{⊤}$.

Then, the distributed optimization problem is transformed into a multi-agent consensus, which implies when $\phi_{i}\left(t\right)=\phi_{j}\left(t\right)$, ∀i,j∈N, the RAP (1) is obtained for any agents. Then, $\phi^{*}$ is the final value of $\phi_{i}\left(t\right)$ when it reaches consensus. The detailed procedure of the one-to-all DET strategy is given as Algorithm 1.
**Algorithm 1** Distributed optimization algorithm with the one-to-all DET strategy**Require:**   Initialize all parameters, such as the states $x_{i}\left(0\right)$ and $\zeta_{i}\left(0\right)$ of the agent *i* and so on. During the initialization process, it is required that $\sum_{i=1}^{n}x_{i}\left(0\right)=D$ and $\zeta_{i}\left(0\right)=0$. Input last triggering times $t_{k}^{i}$ and state ${\hat{\phi }}_{i}\left(t\right)$, ∀i∈N.**Ensure:**   **for** t=0 to $t_{\mathrm{end}}$ **do**    **for** i=1 to *n* **do**      Compute measurement errors with $e_{i}\left(t\right)$.      Compute the trigger threshold $c_{i}\sum_{j=1}^{n}a_{ij}{\left(\phi_{i}\left(t\right)-\phi_{j}\left(t\right)\right)}^{2}-\pi_{i}\Gamma_{i}\left(t\right)$.      **if** trigger condition (2) holds **then**         The event is triggered, and the event time is recorded as $t_{k+1}^{i}$.         Update the state ${\hat{\phi }}_{i}\left(t\right)$ of agent *i* at event time $t_{k+1}^{i}$.         Communicate information between state ${\hat{\phi }}_{i}\left(t\right)$ and its neighbor state ${\hat{\phi }}_{j}\left(t\right)$.      **else**         Update the state ${\hat{\phi }}_{i}\left(t\right)$ of agent *i* at instant *t* which belongs to interval $\left[t_{k}^{i},t_{k+1}^{i}\right)$.      **end if**    **end for** **end for**

**Remark** **2.** For the quadratic original optimization problem with the equality constraint, based on the Lagrange multiplier method, we construct the Lagrangian function as $L\left(x\left(t\right),\lambda \right)=\sum_{i=1}^{n}f_{i}\left(x_{i}\left(t\right)\right)-\lambda \left(\sum_{i=1}^{n}x_{i}\left(t\right)-D\right)$. Then, under Assumption 2, $x_{i}^{*}=\frac{\lambda^{*}-\beta_{i}}{2\alpha_{i}}$, i∈N is the optimal solution, where $\lambda^{*}$ is the optimal Lagrange multipliers if and only if $\frac{\partial f_{1}\left(x_{1}\left(t\right)\right)}{\partial x_{1}\left(t\right)}=\frac{\partial f_{2}\left(x_{2}\left(t\right)\right)}{\partial x_{2}\left(t\right)}=\cdots =\frac{\partial f_{n}\left(x_{n}\left(t\right)\right)}{\partial x_{n}\left(t\right)}=\lambda^{*}$, i∈N. Therefore, we need to let the Lagrange multiplier $\lambda_{i}\in R$ of each agent update $\lambda_{i}$ so that all $\lambda_{i}$ reach consensus at the value $\lambda^{*}$, which means that the optimization problem with equality constraint is transformed to a MASs consensus problem completely. Therefore, as long as the equation $\phi_{1}\left(t\right)=\phi_{2}\left(t\right)=\cdots =\phi_{n}\left(t\right)=\lambda^{*}=\frac{D+\sum_{i=1}^{n}\frac{\beta_{i}}{2\alpha_{i}}}{\sum_{i=1}^{n}\frac{1}{2\alpha_{i}}}$ holds, the algorithm can achieve consensus and the optimization problem can be addressed.

**Remark** **3.** *Algorithm (4) only uses the information of variable $x_{i}\left(t\right)$, which is beneficial to save communication resources in the case of limited bandwidth. Furthermore, let $\zeta \left(t\right)=(\zeta_{1}\left(t\right),\zeta_{2}\left(t\right),\cdots ,$$\zeta_{n}{\left(t\right))}^{⊤}$, from Assumption 1, i.e., $1_{n}^{⊤}L=0_{n}^{⊤}$, the proposed zero-initial-value distributed optimization algorithm, i.e., $\zeta \left(0\right)=0_{n}$, satisfies the equality constraint at all times. The initial values of the algorithm are composed of the decision variable initial value x(0) and the auxiliary variable initial value $\zeta \left(0\right)=0_{n}$. Then, we can prove that ∀t≥0, $\sum_{i=1}^{n}\zeta_{i}\left(t\right)=\sum_{i=1}^{n}\zeta_{i}\left(0\right)=0$ and $\sum_{i=1}^{n}x_{i}\left(t\right)=\sum_{i=1}^{n}x_{i}\left(0\right)$, because the equation $\sum_{i=1}^{n}{\dot{\zeta }}_{i}\left(t\right)=1_{n}^{⊤}L\nabla f\left(x\left(t\right)\right)=0$ holds. Therefore, when the equation $\sum_{i=1}^{n}x_{i}\left(0\right)=D$ is satisfied, the equality constraint holds as well at any time.*

**Theorem** **1.** *Under Assumptions 1 and 2, assume that $\psi_{i}\geq \pi_{i}\left(2-\frac{w_{i}}{c_{i}}\right)$, $0<c_{i}\leq \frac{1}{4}$, then the RAP (1) is solved under the distributed optimization algorithm (4) and the DET strategies (2) and (3). Moreover, Zeno behavior is excluded.*

**Proof.**  Construct the Lyapunov function $W_{1}\left(t\right)$ of the following form

$$\begin{array}{cc} W_{1}\left(t\right) & =U\left(t\right)+\sum_{i=1}^{n}\Gamma_{i}\left(t\right), \end{array}$$

where $U\left(t\right)=\frac{1}{2}\sum_{i=1}^{n}\frac{1}{\alpha_{i}}{\left(\phi_{i}\left(t\right)-\phi^{*}\right)}^{2}.$    □

The rest of the proof is the similar to Theorem 2.

## 4. The One-to-One DET Strategy

In this section, in consideration of the existence of asynchronous transmission needs, the one-to-one DET strategy is introduced, which has the unique characteristics that each agent transmits its information to all of its neighbors asynchronously, unlike the one-to-all DET strategy. Furthermore, based on the one-to-one DET strategy, a more flexible distributed optimization algorithm is similarly presented and the consensus is achieved, which also solves the RAP (1). Then, we prove that the Zeno behavior will not occur, which strongly ensures that the algorithm is implementable.

For the one-to-one DET strategy, the edge-dependent triggering time sequence is raised, i.e., $0=t_{0}^{i\to j}<t_{1}^{i\to j}<\cdots <t_{s}^{i\to j}<\cdots$, which essentially differs from the one-to-all case.

Corresponding to the one-to-one DET case, the measurement error is described as

$$\begin{array}{cc} e_{i}^{j}\left(t\right) & =\nabla f_{i}\left(x_{i}\left(t_{k}^{i\to j}\right)\right)-\nabla f_{i}\left(x_{i}\left(t\right)\right),\forall i\in N,j\in N_{i}. \end{array}$$

Then, we propose the one-to-one DET triggering sequence ${\left\{t_{k}^{i\to j}\right\}}_{k\in N}$ as follows

(5a) $$\begin{array}{c} t_{k+1}^{i\to j}=\underset{l>t_{k}^{i\to j}}{\inf}\left\{l:{\left(e_{i}^{j}\left(t\right)\right)}^{2}-c_{ij}{\left(\phi_{i}\left(t\right)-\phi_{j}\left(t\right)\right)}^{2}-\pi_{ij}{\tilde{\Gamma }}_{ij}\left(t\right)\geq 0,\forall t\in \left[t_{k}^{i\to j},l\right]\right\}, \end{array}$$

where $c_{ij}$ and $\pi_{ij}$, $\forall i\in N,j\in N_{i}$, are positive constants.

**Remark** **4.** *Similarly, if setting ${\tilde{\Gamma }}_{ij}\left(t\right)=0$, the DET strategy reduces to the SET strategy. Then, the one-to-one SET triggering sequence ${\left\{t_{k}^{i\to j}\right\}}_{k\in N}$ is as follows*

(5b) $$\begin{array}{c} t_{k+1}^{i\to j}=\underset{l>t_{k}^{i\to j}}{\inf}\left\{l:{\left(e_{i}^{j}\left(t\right)\right)}^{2}-c_{ij}{\left(\phi_{i}\left(t\right)-\phi_{j}\left(t\right)\right)}^{2}\geq 0,\forall t\in \left[t_{k}^{i\to j},l\right]\right\}. \end{array}$$

Inspired by [43], we design an internal dynamic variable ${\tilde{\Gamma }}_{ij}\left(t\right)$ satisfying

(6) $$\begin{array}{c} {\dot{\tilde{\Gamma }}}_{ij}\left(t\right)=-{\tilde{\psi }}_{ij}{\tilde{\Gamma }}_{ij}\left(t\right)+{\tilde{\mu }}_{ij}\left[c_{ij}{\left(\phi_{i}\left(t\right)-\phi_{j}\left(t\right)\right)}^{2}-{\left(e_{i}^{j}\left(t\right)\right)}^{2}\right], \end{array}$$

where ${\tilde{\Gamma }}_{ij}\left(0\right)>0$, ${\tilde{\psi }}_{ij}$ and ${\tilde{\mu }}_{ij}=\frac{{\tilde{w}}_{ij}}{c_{ij}}$$\mathrm{with}\;{\tilde{w}}_{ij}\geq 0,$$\;\forall i\in N,j\in N_{i}$, are positive constants. In addition, ${\dot{\tilde{\Gamma }}}_{ij}\left(t\right)\geq -\left({\tilde{\psi }}_{ij}+\frac{{\tilde{w}}_{ij}}{c_{ij}}\right){\tilde{\Gamma }}_{ij}\left(t\right)$, ∀t>0, and thus ${\tilde{\Gamma }}_{ij}\left(t\right)\geq {\tilde{\Gamma }}_{ij}\left(0\right)\exp\left(-\left({\tilde{\psi }}_{ij}+\frac{{\tilde{w}}_{ij}}{c_{ij}}\right)t\right)>0$.

Let $\hat{\phi_{i}^{j}}\left(t\right)=\nabla f_{i}\left(x_{i}\left(t_{k}^{i\to j}\right)\right)$. The distributed optimization algorithm is determined as follows to solve the RAP (1):

(7) $$\begin{array}{c} \left\{\begin{array}{c} x_{i}\left(t\right)=\zeta_{i}\left(t\right)+x_{i}\left(0\right),\\ {\dot{\zeta }}_{i}\left(t\right)=\sum_{j=1}^{n}a_{ij}\left(\hat{\phi_{j}^{i}}\left(t\right)-\hat{\phi_{i}^{j}}\left(t\right)\right),\\ \zeta_{i}\left(0\right)=0, \end{array}\right. \end{array}$$

for $\forall i\in N,j\in N_{i}$. In addition, one obtains

(8) $$\begin{array}{cc} {\dot{\phi }}_{i}\left(t\right) & =2\alpha_{i}\sum_{j=1}^{n}a_{ij}\left[\hat{\phi_{j}^{i}}\left(t\right)-\hat{\phi_{i}^{j}}\left(t\right)\right]\\ & =2\alpha_{i}\sum_{j=1}^{n}a_{ij}\left[\left(\phi_{j}\left(t\right)+e_{j}^{i}\left(t\right)\right)-\left(\phi_{i}\left(t\right)+e_{i}^{j}\left(t\right)\right)\right], \end{array}$$

where the initial value $\phi_{i}\left(0\right)$, ∀i∈N, satisfies the equation $\phi_{i}\left(0\right)=2\alpha_{i}x_{i}\left(0\right)+\beta_{i}.$ The detailed one-to-one DET procedure is given as Algorithm 2.

**Theorem** **2.** *Under Assumptions 1 and 2, if the parameters ${\tilde{\psi }}_{ij}$ and $c_{ij}$ in (5a,b) and (6) satisfy ${\tilde{\psi }}_{ij}\geq \pi_{ij}\left(2-\frac{{\tilde{w}}_{ij}}{c_{ij}}\right)$, $0<c_{ij}\leq \frac{1}{4}$, then the RAP (1) is solved under the distributed optimization algorithm (7) and the DET strategies (5a,b) and (6). Moreover, Zeno behavior is excluded.*

**Algorithm 2** Distributed optimization algorithm with the one-to-one DET strategy
**Require:**   Initialize all parameters, such as the states $x_{i}\left(0\right)$ and $\zeta_{i}\left(0\right)$ of the agent *i* and so on. During the initialization process, it is required that $\sum_{i=1}^{n}x_{i}\left(0\right)=D$ and $\zeta_{i}\left(0\right)=0$. Input last triggering times $t_{k}^{i\to j}$ and state ${\hat{\phi }}_{i}^{j}\left(t\right)$, ∀i∈N.**Ensure:**   **for** t=0 to $t_{\mathrm{end}}$ **do**    **for** i=1 to *n* **do**      Compute measurement errors with $e_{i}^{j}\left(t\right)$.      Compute the triggered threshold $c_{ij}{\left(\phi_{i}\left(t\right)-\phi_{j}\left(t\right)\right)}^{2}-\pi_{ij}{\tilde{\Gamma }}_{ij}\left(t\right)$.      **if** trigger condition (5a,b) holds **then**         The event is triggered, and the event time is recorded as $t_{k+1}^{i\to j}$.         Update the state ${\hat{\phi }}_{i}^{j}\left(t\right)$ of agent *i* to agent *j* at event time $t_{k+1}^{i\to j}$.         Communicate information between state ${\hat{\phi }}_{i}^{j}\left(t\right)$ and its neighbor state ${\hat{\phi }}_{j}^{i}\left(t\right)$.      **else**         Update the state ${\hat{\phi }}_{i}^{j}\left(t\right)$ of agent *i* at instant *t* which belongs to interval $\left[t_{k}^{i\to j},t_{k+1}^{i\to j}\right)$.      **end if**    **end for** 
**end for**

**Proof.** 

 

(i) Define the following Lyapunov function:

$$W\left(t\right)=V\left(t\right)+\sum_{i=1}^{n}\sum_{j=1}^{n}a_{ij}{\tilde{\Gamma }}_{ij}\left(t\right),$$

where $V\left(t\right)=\frac{1}{2}\sum_{i=1}^{n}\frac{1}{\alpha_{i}}{\left(\phi_{i}\left(t\right)-\phi^{*}\right)}^{2}.$

From (8), we have

(9) $$\begin{array}{cc} \dot{V}\left(t\right) & =\sum_{i=1}^{n}\frac{1}{\alpha_{i}}\left(\phi_{i}\left(t\right)-\phi^{*}\right){\dot{\phi }}_{i}\left(t\right)\\ \; & =\sum_{i=1}^{n}\frac{1}{\alpha_{i}}\left(\phi_{i}\left(t\right)-\phi^{*}\right)2\alpha_{i}\sum_{j=1}^{n}a_{ij}\left[\left(\phi_{j}\left(t\right)+e_{j}^{i}\left(t\right)\right)-\left(\phi_{i}\left(t\right)+e_{i}^{j}\left(t\right)\right)\right]\\ & =\sum_{i=1}^{n}\sum_{j=1}^{n}2a_{ij}\left(\phi_{i}\left(t\right)-\phi^{*}\right)\left[\left(\phi_{j}\left(t\right)+e_{j}^{i}\left(t\right)\right)-\left(\phi_{i}\left(t\right)+e_{i}^{j}\left(t\right)\right)\right]\\ & =\sum_{i=1}^{n}\sum_{j=1}^{n}2a_{ij}\phi_{i}\left(t\right)\left[\left(\phi_{j}\left(t\right)+e_{j}^{i}\left(t\right)\right)-\left(\phi_{i}\left(t\right)+e_{i}^{j}\left(t\right)\right)\right]\\ & =\sum_{i=1}^{n}\sum_{j=1}^{n}2a_{ij}\left[\phi_{i}\left(t\right)\left(\phi_{j}\left(t\right)-\phi_{i}\left(t\right)\right)+\phi_{i}\left(t\right)\left(e_{j}^{i}\left(t\right)-e_{i}^{j}\left(t\right)\right)\right]. \end{array}$$

Note that

(10) $$\begin{array}{cc} \; & \;\sum_{i=1}^{n}\sum_{j=1}^{n}2a_{ij}\phi_{i}\left(t\right)\left(\phi_{j}\left(t\right)-\phi_{i}\left(t\right)\right)\\ \; & =\sum_{i=1}^{n}\sum_{j=1}^{n}a_{ij}\left[\phi_{i}\left(t\right)\left(\phi_{j}\left(t\right)-\phi_{i}\left(t\right)\right)+\phi_{j}\left(t\right)\left(\phi_{i}\left(t\right)-\phi_{j}\left(t\right)\right)\right]\\ \; & =-\sum_{i=1}^{n}\sum_{j=1}^{n}a_{ij}{\left[\phi_{i}\left(t\right)-\phi_{j}\left(t\right)\right]}^{2}. \end{array}$$

From Young’s inequality, one has

(11) $$\begin{array}{cc} \; & \;\sum_{i=1}^{n}\sum_{j=1}^{n}2a_{ij}\phi_{i}\left(t\right)\left(e_{j}^{i}\left(t\right)-e_{i}^{j}\left(t\right)\right)\leq \sum_{i=1}^{n}\sum_{j=1}^{n}a_{ij}\left[2{\left(e_{i}^{j}\left(t\right)\right)}^{2}+\frac{1}{2}{\left(\phi_{i}\left(t\right)-\phi_{j}\left(t\right)\right)}^{2}\right]. \end{array}$$

Substituting (10) and (11) into (9) yields

(12) $$\begin{array}{c} \dot{V}\left(t\right)\leq \sum_{i=1}^{n}\sum_{j=1}^{n}a_{ij}\left[2{\left(e_{i}^{j}\left(t\right)\right)}^{2}-\frac{1}{2}{\left(\phi_{i}\left(t\right)-\phi_{j}\left(t\right)\right)}^{2}\right]. \end{array}$$

According to Formula (12), taking the derivative of the Lyapunov function W(t) can be derived as

$$\dot{W}\left(t\right)\leq \sum_{i=1}^{n}\sum_{j=1}^{n}a_{ij}\left[2{\left(e_{i}^{j}\left(t\right)\right)}^{2}-\frac{1}{2}{\left(\phi_{i}\left(t\right)-\phi_{j}\left(t\right)\right)}^{2}+{\dot{\tilde{\Gamma }}}_{ij}\left(t\right)\right].$$

Then, we can obtain from (5a,b) and (6) that

$$\begin{array}{cc} \dot{W}\left(t\right) & \leq \sum_{i=1}^{n}\sum_{j=1}^{n}a_{ij}[2{\left(e_{i}^{j}\left(t\right)\right)}^{2}-\frac{1}{2}{\left(\phi_{i}\left(t\right)-\phi_{j}\left(t\right)\right)}^{2}-{\tilde{\psi }}_{ij}{\tilde{\Gamma }}_{ij}\left(t\right)\\ \; & \;+{\tilde{w}}_{ij}{\left(\phi_{i}\left(t\right)-\phi_{j}\left(t\right)\right)}^{2}-{\tilde{\mu }}_{ij}{\left(e_{i}^{j}\left(t\right)\right)}^{2}]\\ \; & \leq \sum_{i=1}^{n}\sum_{j=1}^{n}a_{ij}\left[\left(2-\frac{{\tilde{w}}_{ij}}{c_{ij}}\right){\left(e_{i}^{j}\left(t\right)\right)}^{2}+\left({\tilde{w}}_{ij}-\frac{1}{2}\right){\left(\phi_{i}\left(t\right)-\phi_{j}\left(t\right)\right)}^{2}-{\tilde{\psi }}_{ij}{\tilde{\Gamma }}_{ij}\left(t\right)\right]\\ \; & \leq \sum_{i=1}^{n}\sum_{j=1}^{n}a_{ij}[\left(2-\frac{{\tilde{w}}_{ij}}{c_{ij}}\right)\left(c_{ij}{\left(\phi_{i}\left(t\right)-\phi_{j}\left(t\right)\right)}^{2}+\pi_{ij}{\tilde{\Gamma }}_{ij}\left(t\right)\right)\\ \; & \;+\left({\tilde{w}}_{ij}-\frac{1}{2}\right){\left(\phi_{i}\left(t\right)-\phi_{j}\left(t\right)\right)}^{2}-{\tilde{\psi }}_{ij}{\tilde{\Gamma }}_{ij}\left(t\right)]\\ \; & =\sum_{i=1}^{n}\sum_{j=1}^{n}a_{ij}\left[\left(2c_{ij}-\frac{1}{2}\right){\left(\phi_{i}\left(t\right)-\phi_{j}\left(t\right)\right)}^{2}+\left(\left(2-\frac{{\tilde{w}}_{ij}}{c_{ij}}\right)\pi_{ij}-{\tilde{\psi }}_{ij}\right){\tilde{\Gamma }}_{ij}\left(t\right)\right]. \end{array}$$

Since ${\tilde{\psi }}_{ij}\geq \pi_{ij}\left(2-\frac{{\tilde{w}}_{ij}}{c_{ij}}\right)$, $\pi_{ij}>0$, ${\tilde{\mu }}_{ij}=\frac{{\tilde{w}}_{ij}}{c_{ij}}\;\mathrm{and}\;0<c_{ij}\leq \frac{1}{4}$, one obtains $\dot{W}\left(t\right)\leq 0$. This implies that W(t) cannot increase and that $\phi_{i}\left(t\right)-\phi_{j}\left(t\right)$ and ${\tilde{\Gamma }}_{ij}\left(t\right)$ are bounded. In addition, ${\tilde{\Gamma }}_{ij}\left(t\right)>0$, ∀t≥0, which leads to W(t)>0.

By LaSalle’s invariance principle in [44], one obtains $\lim_{t\to \infty }\left|\phi_{i}\left(t\right)-\phi_{j}\left(t\right)\right|=0$, $\lim_{t\to \infty }{\tilde{\Gamma }}_{ij}\left(t\right)=0\left(\forall i,j\in E\right).$ Thus, the RAP (1) is solved eventually.

(ii) In this part, we prove that Zeno behavior does not occur by contradiction. Assume that the triggering sequence ${\left\{t_{k}^{i\to j}\right\}}_{k\in N}$ determined by (7) and (8) leads to Zeno behavior, which indicates that for any $\varepsilon^{*}>0$ there exists a $K\left(\varepsilon^{*}\right)\in Z^{+}$ such that for any $k\geq K(\varepsilon^{*})$, $|t_{k}^{i\to j}-t_{*}^{i\to j}|<\varepsilon^{*}.$

Evidently,

(13) $$\begin{array}{c} t_{K(\varepsilon^{*})+1}^{i\to j}-t_{K(\varepsilon^{*})}^{i\to j}<2\varepsilon^{*}. \end{array}$$

For $\forall \;t\in [0,T_{*}^{ij}]$, $\exists \;\varepsilon_{1}$, $\varepsilon_{2}>0,\;s.t.\;\left|\phi_{i}\left(t\right)-\phi_{j}\left(t\right)\right|\leq \varepsilon_{1},$$|{\tilde{\Gamma }}_{ij}\left(t\right)|\leq \varepsilon_{2}$.

Then, for $t_{k}^{i\to j}\leq t\leq t_{k+1}^{i\to j}$, from (8),

$$\begin{array}{cc} D^{+}\left|e_{i}^{j}\left(t\right)\right| & \leq |{\dot{e}}_{i}^{j}\left(t\right)|=|{\dot{\phi }}_{i}\left(t\right)|\\ \; & =|2\alpha_{i}\sum_{j=1}^{n}a_{ij}\left(\hat{\phi_{j}^{i}}\left(t\right)-\hat{\phi_{i}^{j}}\left(t\right)\right)|\\ \; & \leq 2\bar{\alpha }\Pi_{k}^{ij}, \end{array}$$

where $\bar{\alpha }=\max_{1\leq i\leq n}\left\{\alpha_{i}\right\}$ and $\Pi_{k}^{ij}=\max_{t\in [t_{k}^{i\to j},t_{k+1}^{i\to j})}\{|\sum_{j=1}^{n}a_{ij}\left({\hat{\phi }}_{j}^{i}\left(t\right)-{\hat{\phi }}_{i}^{j}\left(t\right)\right)|\}$.

Therefore, for any $t\in \left[t_{k}^{i\to j},t_{k+1}^{i\to j}\right)$,

(14) $${\left(e_{i}^{j}\left(t\right)\right)}^{2}\leq 4{\bar{\alpha }}^{2}{\Pi_{k}^{ij}}^{2}{\left(t-t_{k}^{i\to j}\right)}^{2}.$$

By the trigger conditions (5a,b) and (6), when $t=t_{k+1}^{i\to j}$,

$$\begin{array}{cc} |e_{i}^{j}\left(t_{k+1}^{i\to j}\right)| & =\sqrt{c_{ij}}\left|\phi_{i}\left(t_{k+1}^{i\to j}\right)-\phi_{j}\left(t_{k+1}^{i\to j}\right)\right|+\sqrt{\pi_{ij}{\tilde{\Gamma }}_{ij}\left(t_{k+1}^{i\to j}\right)}\\ & \geq \sqrt{\pi_{ij}{\tilde{\Gamma }}_{ij}\left(t_{k+1}^{i\to j}\right)}\\ & >0. \end{array}$$

Noting

$$\begin{array}{cc} {\dot{\tilde{\Gamma }}}_{ij}\left(t\right) & =-{\tilde{\psi }}_{ij}{\tilde{\Gamma }}_{ij}\left(t\right)+{\tilde{\mu }}_{ij}\left[c_{ij}{\left(\phi_{i}\left(t\right)-\phi_{j}\left(t\right)\right)}^{2}-{\left(e_{i}^{j}\left(t\right)\right)}^{2}\right]\\ & \geq -{\tilde{\psi }}_{ij}{\tilde{\Gamma }}_{ij}\left(t\right)-\pi_{ij}{\tilde{\Gamma }}_{ij}\left(t\right)\\ & =-\left({\tilde{\psi }}_{ij}+\pi_{ij}\right){\tilde{\Gamma }}_{ij}\left(t\right). \end{array}$$

By using the comparison principle,

$$\begin{array}{cc} \; & {\tilde{\Gamma }}_{ij}\left(t\right)\geq {\tilde{\Gamma }}_{ij}\left(0\right)e^{-({\tilde{\psi }}_{ij}+\pi_{ij})t}, \end{array}$$

$$\begin{array}{c} {\tilde{\Gamma }}_{ij}\left(t_{k+1}^{i\to j}\right)\geq {\tilde{\Gamma }}_{ij}\left(0\right)e^{-\left({\tilde{\psi }}_{ij}+\pi_{ij}\right)t_{k+1}^{i\to j}}, \end{array}$$

$$\begin{array}{c} |e_{i}^{j}\left(t_{k+1}^{i\to j}\right)|\geq \sqrt{\pi_{ij}{\tilde{\Gamma }}_{ij}\left(t_{k+1}^{i\to j}\right)}\geq \sqrt{\pi_{ij}{\tilde{\Gamma }}_{ij}\left(0\right)e^{-\left({\tilde{\psi }}_{ij}+\pi_{ij}\right)t_{k+1}^{i\to j}}}, \end{array}$$

(15) $$\begin{array}{c} {\left(e_{i}^{j}\left(t_{k+1}^{i\to j}\right)\right)}^{2}\geq \pi_{ij}{\tilde{\Gamma }}_{ij}\left(0\right)e^{-\left({\tilde{\psi }}_{ij}+\pi_{ij}\right)t_{k+1}^{i\to j}}. \end{array}$$

Combining (14) and (15), it has

$$\pi_{ij}{\tilde{\Gamma }}_{ij}\left(0\right)e^{-\left({\tilde{\psi }}_{ij}+\pi_{ij}\right)t_{k+1}^{i\to j}}\leq 4{\bar{\alpha }}^{2}{\Pi_{k}^{ij}}^{2}{\left(t_{k+1}^{i\to j}-t_{k}^{i\to j}\right)}^{2}.$$

Therefore,

(16) $$\begin{array}{cc} t_{k+1}^{i\to j}-t_{k}^{i\to j} & \geq \frac{\sqrt{\pi_{ij}{\tilde{\Gamma }}_{ij}\left(0\right)e^{-\left({\tilde{\alpha }}_{ij}+\pi_{ij}\right)t_{k+1}^{i\to j}}}}{2\bar{\alpha }\Pi_{k}^{ij}}\\ \; & \geq \frac{\sqrt{\pi_{ij}{\tilde{\Gamma }}_{ij}\left(0\right)e^{-\left({\tilde{\alpha }}_{ij}+\pi_{ij}\right)T_{*}^{ij}}}}{2\bar{\alpha }\Pi_{k}^{ij}}. \end{array}$$

For $\varepsilon^{*}=\frac{\sqrt{\pi_{ij}{\tilde{\Gamma }}_{ij}\left(0\right)e^{-\left({\tilde{\alpha }}_{ij}+\pi_{ij}\right)T_{*}^{ij}}}}{4\bar{\alpha }\Pi_{k}^{ij}}>0,$ it is not difficult to see from (16) that $t_{K(\varepsilon^{*})+1}^{i\to j}-t_{K(\varepsilon^{*})}^{i\to j}\geq 2\varepsilon^{*}$, which is obviously contradictory to (13). Consequently, there is no Zeno behavior. □

**Remark** **5.** *In contrast to the one-to-all DET strategy mentioned in Theorem 1, under the one-to-one DET strategy, the triggering sequences $\left\{t_{k}^{i\to j}\right\}\left(j\in N_{i}\right)$ of each agent is different, which contributes to flexibly adjusting the transferred information to each of its neighbors $j\in N_{i}$. Furthermore, the remarkable feature of the one-to-one DET strategy is that each agent is allowed to design its own distinctive triggering instant $t_{k}^{i\to j}$ which is immune to any synchronous executions and the requirements of $t_{k}^{i\to j}=t_{k}^{j\to i}$ or $t_{k}^{i\to j_{1}}=t_{k}^{i\to j_{2}}(\forall j_{1}$, $j_{2}\in N_{i})$, and so on. Therefore, in practice, one-to-one DET strategies potentially offer greater flexibility and efficiency in terms of adjusting the transmission of information, which is significant to designing a good DET strategy.*

**Remark** **6.** *The proposed algorithms (4) and (7) can effectively solve RAP, but both of them need to satisfy $\sum_{i=1}^{n}x_{i}\left(0\right)=D$ and $\zeta_{i}\left(0\right)=0$, which means with initialization constraints. In our future research, we will consider eliminating state initialization.*

## 5. Numerical Example

**Figure 1:**
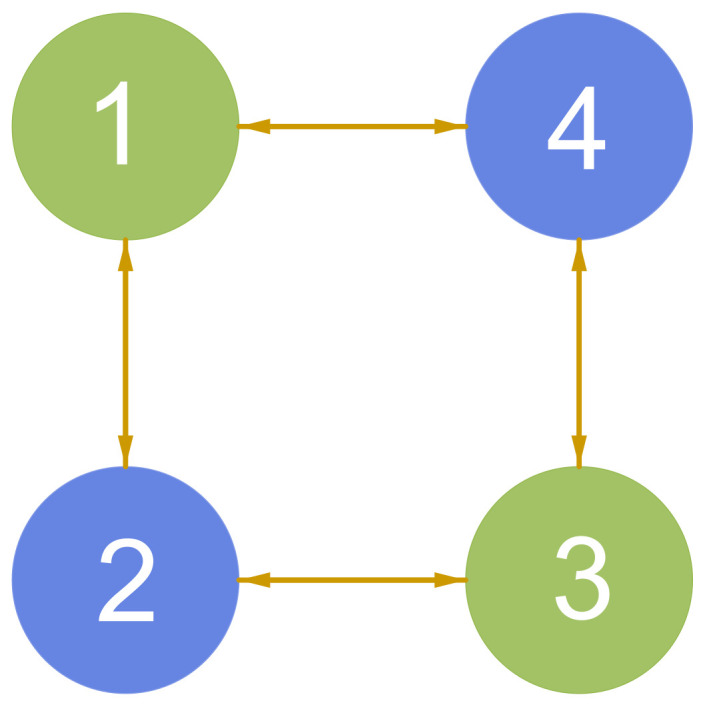
Connection topology.

In this section, two numerical examples are provided to illustrate the effectiveness of the theoretical results. The proposed one-to-all and one-to-one DET strategies are applied to the RAP (1) in case 1 and case 2, respectively. Figure 1 depicts the connection topology, which satisfies Assumption 1. The chosen cost coefficients $\alpha_{i}$, $\beta_{i}$, and $\gamma_{i}$ of the quadratic cost function $f_{i}\left(x_{i}\left(t\right)\right)=\alpha_{i}x_{i}^{2}\left(t\right)+\beta_{i}x_{i}\left(t\right)+\gamma_{i}$ are listed in Table 2. The load demand *D* is assumed to be 145. Then, the initial values of $x_{i}\left(t\right)$ are selected as $x_{1}\left(0\right)=30$, $x_{2}\left(0\right)=25$, $x_{3}\left(0\right)=40$, $x_{4}\left(0\right)=50$, and $\zeta_{i}\left(0\right)=0$, i=1,2,3,4.

**Case 1. One-to-all DET**

**Figure 2:**
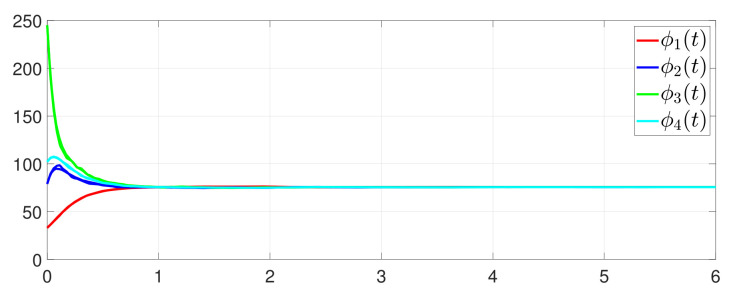
Trajectory of $\phi_{i}\left(t\right)$.

**Figure 3:**
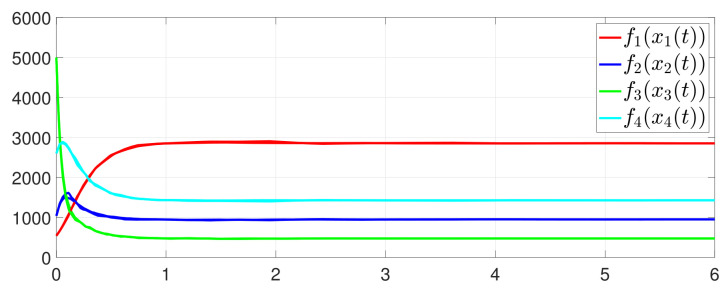
State evolution of $f_{i}\left(x_{i}\left(t\right)\right)$.

**Figure 4:**
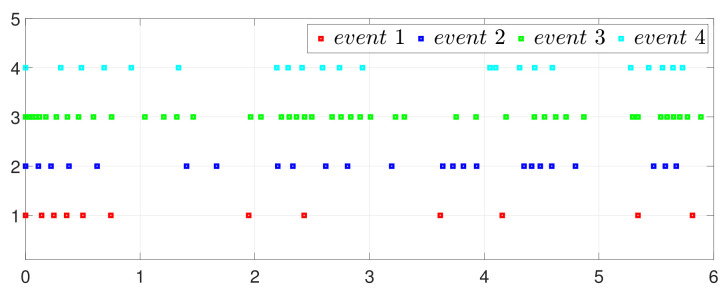
Event triggering instants under one-to-all DET.

**Figure 5:**
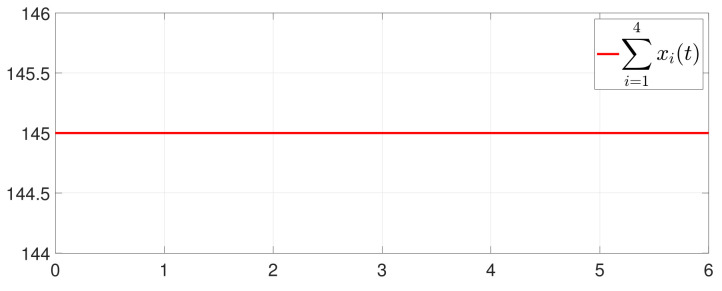
Trajectory of $\sum_{i=1}^{4}x_{i}\left(t\right)$.

First, consider the one-to-all DET based on Theorem 1. Given the scalars $\Gamma_{1}\left(0\right)=4$, $\Gamma_{2}\left(0\right)=3$, $\Gamma_{3}\left(0\right)=8$, $\Gamma_{4}\left(0\right)=6$, $\;c_{1}=0.05$, $c_{2}=0.1$, $c_{3}=0.15$, $c_{4}=0.2$, $\omega_{1}=0.025$, $\omega_{2}=0.03$, $\omega_{3}=0.06$, $\omega_{4}=0.07$, $\mu_{1}=0.5$, $\mu_{2}=0.3$, $\mu_{3}=0.4$, $\mu_{4}=0.35$, $\pi_{1}=1$, $\pi_{2}=2$, $\pi_{3}=3$, $\pi_{4}=4$, $\psi_{1}=1.5$, $\psi_{2}=3.4$, $\psi_{3}=4.8$, $\psi_{4}=6.6$, Figure 2 shows that $\phi_{i}\left(t\right)$ converges to $\phi^{*}$, which essentially guarantees that all agents reach asymptotic consensus. Then, from Figure 3, $f_{i}\left(x_{i}\left(t\right)\right)$ converges to the optimal values. Figure 4 shows the triggering instants of the one-to-all DET strategy. In addition, the equality constraint $\sum_{i=1}^{4}x_{i}\left(t\right)=D$ can be obtained from Figure 5.

**Figure 6:**
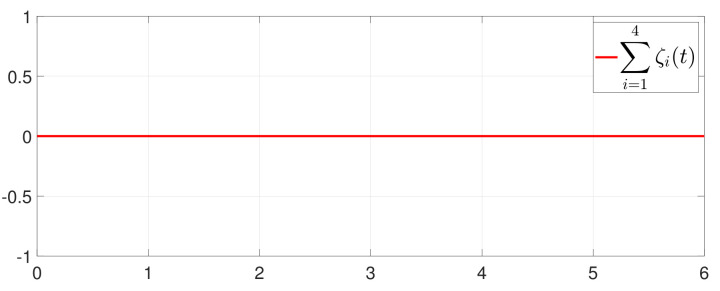
Trajectory of $\sum_{i=1}^{4}\zeta_{i}\left(t\right)$.

**Figure 7:**
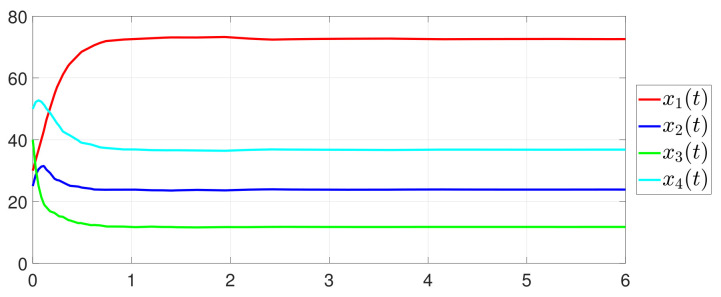
State evolution of $x_{i}\left(t\right)$.

**Figure 8:**
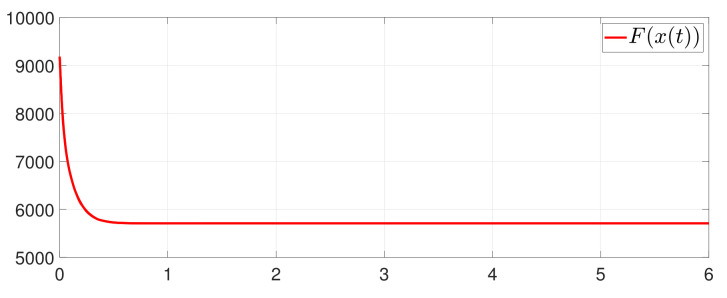
State evolution of F(x(t)).

**Figure 9:**
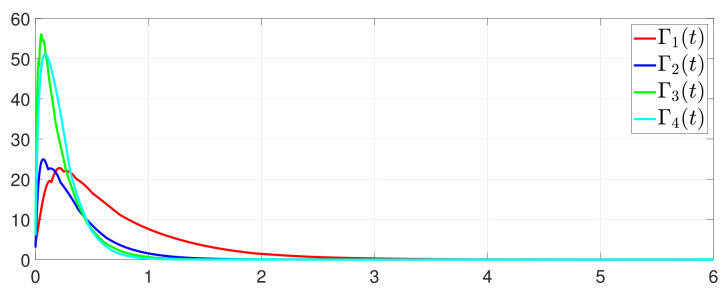
State evolution of $\Gamma_{i}\left(t\right)$.

Furthermore, the equality constraint $\sum_{i=1}^{4}\zeta_{i}\left(t\right)=0$ can be obtained from Figure 6. The trajectories of $x_{i}\left(t\right)$ are shown in Figure 7. Moreover, Figure 8 shows the minimum value of F(x(t)), where $F(x^{*})$ is the optimal solution of the RAP (1). Figure 9 exhibits the trajectory of the dynamic variable $\Gamma_{i}\left(t\right)$.

**Case 2. One-to-one DET**

**Figure 10:**
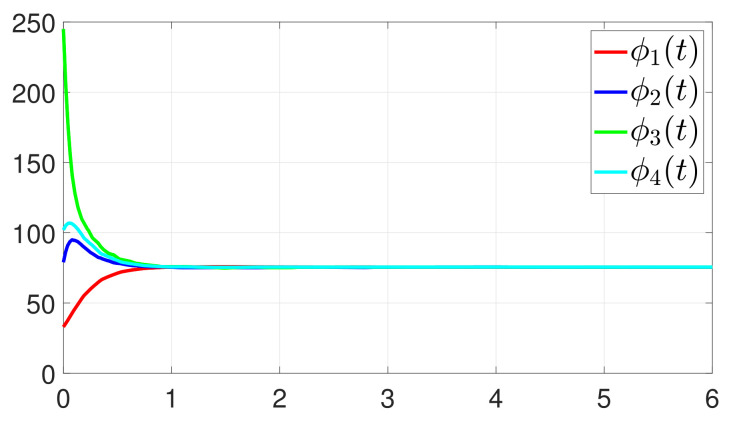
Trajectory of $\phi_{i}\left(t\right)$.

**Figure 11:**
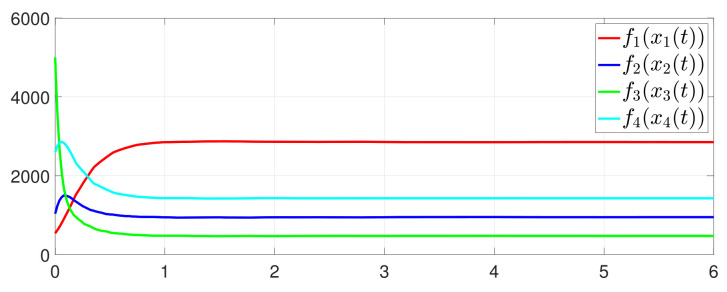
State evolution of $f_{i}\left(x_{i}\left(t\right)\right)$.

**Figure 12:**
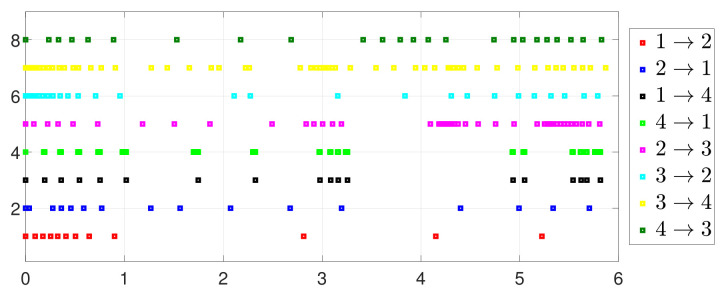
Event triggering instants under one-to-one DET.

**Figure 13:**
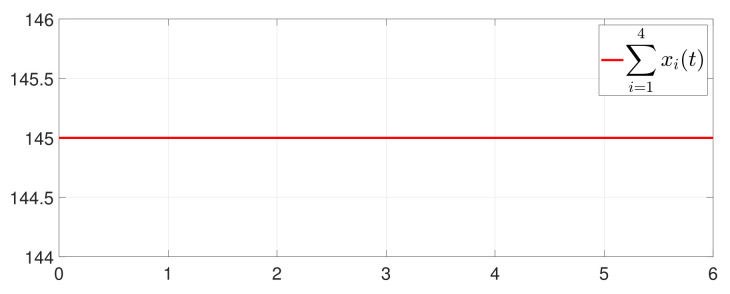
Trajectory of $\sum_{i=1}^{4}x_{i}\left(t\right)$.

Consider the one-to-one DET based on Theorem 2. Different from case 1, given ${\tilde{\Gamma }}_{12}\left(0\right)=1,\;{\tilde{\Gamma }}_{23}\left(0\right)=4,\;{\tilde{\Gamma }}_{34}\left(0\right)=5,\;{\tilde{\Gamma }}_{41}\left(0\right)=10,\;{\tilde{\Gamma }}_{21}\left(0\right)=1,\;{\tilde{\Gamma }}_{32}\left(0\right)=4,\;{\tilde{\Gamma }}_{43}\left(0\right)=5,$ ${\tilde{\Gamma }}_{14}\left(0\right)=10,\;c_{12}=0.05,\;c_{23}=0.1,\;c_{34}=0.15,\;c_{41}=0.2,\;c_{21}=0.04,\;c_{32}=0.08,\;c_{43}=0.12,$ $\;c_{14}=0.24,\;\omega_{12}=0.025,\;\omega_{23}=0.03,\;\omega_{34}=0.06,\;\omega_{41}=0.07,\;\omega_{21}=0.01,\;\omega_{32}=0.05,$ $\;\omega_{43}=0.08,\;\omega_{14}=0.09,\;{\tilde{\mu }}_{12}=0.5,\;{\tilde{\mu }}_{23}=0.3,\;{\tilde{\mu }}_{34}=0.4,\;{\tilde{\mu }}_{41}=0.35,\;{\tilde{\mu }}_{21}=0.25,$ $\;{\tilde{\mu }}_{32}=0.625,\;{\tilde{\mu }}_{43}=0.67,\;{\tilde{\mu }}_{14}=0.375,\;\pi_{12}=1,\;\pi_{23}=2,\;\pi_{34}=3,\;\pi_{41}=4,\;\pi_{21}=1,$ $\;\pi_{32}=2,\;\pi_{43}=3,\;\pi_{14}=4,\;{\tilde{\psi }}_{12}=1.5,\;{\tilde{\psi }}_{23}=3.4,\;{\tilde{\psi }}_{34}=4.8,\;{\tilde{\psi }}_{41}=6.6,\;{\tilde{\psi }}_{21}=1.75,$ $\;{\tilde{\psi }}_{32}=2.75,\;{\tilde{\psi }}_{43}=3.99,\;{\tilde{\psi }}_{14}=6.5$, Figure 10 shows that $\phi_{i}\left(t\right)$ converges to $\phi^{*}$, which also implies that consensus is indeed achieved. Then, as seen in Figure 11, $f_{i}\left(x_{i}\left(t\right)\right)$ converges to the minimum value. Figure 12 shows the triggering instants of the one-to-one DET strategy. In addition, the equation constraint $\sum_{i=1}^{4}x_{i}\left(t\right)=D$ is guaranteed from Figure 13.

**Figure 14:**
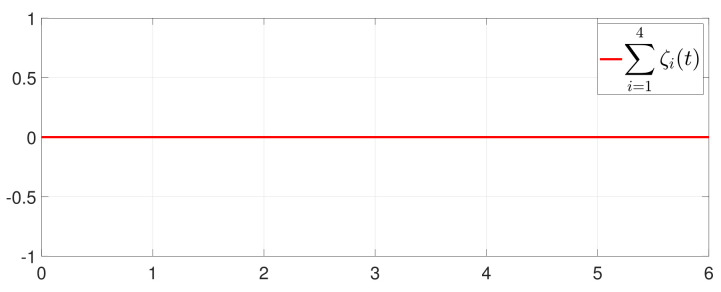
Trajectory of $\sum_{i=1}^{4}\zeta_{i}\left(t\right)$.

**Figure 15:**
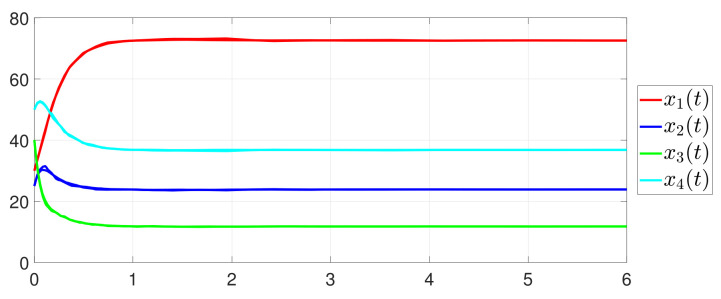
State evolution of $x_{i}\left(t\right)$.

**Figure 16:**
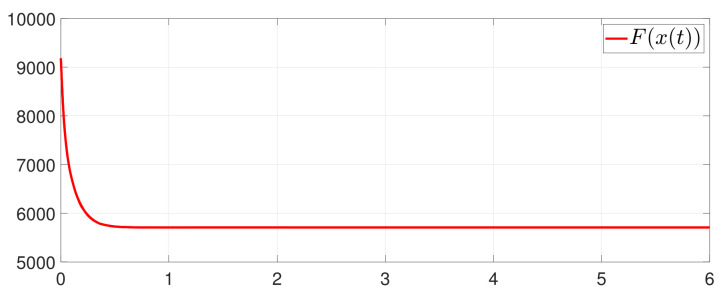
State evolution of F(x(t)).

**Figure 17:**
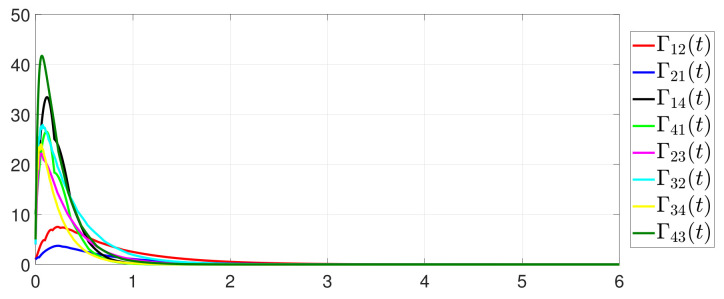
State evolution of ${\tilde{\Gamma }}_{ij}\left(t\right)$.

Besides, the equation constraint $\sum_{i=1}^{4}\zeta_{i}\left(t\right)=0$ is guaranteed from Figure 14. The motion trajectory of $x_{i}\left(t\right)$ is shown in Figure 15. Furthermore, Figure 16 depicts the minimum value of F(x(t)), where $F(x^{*})$ is the optimal solution of the RAP (1). Figure 17 shows that ${\tilde{\Gamma }}_{ij}\left(t\right)$ converges to 0 and ${\tilde{\Gamma }}_{ij}\left(t\right)>0$ always holds.

**Case 3. DET vs. SET**

**Figure 18:**
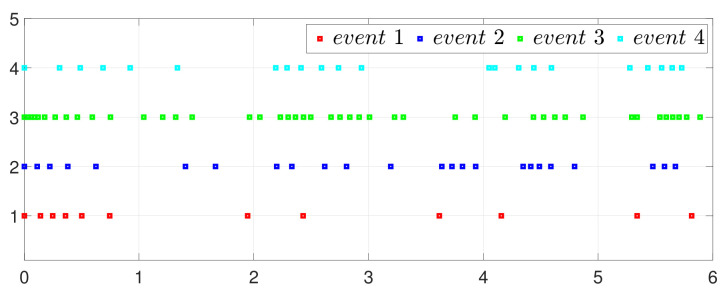
Event under one-to-all DET (2a).

**Figure 19:**
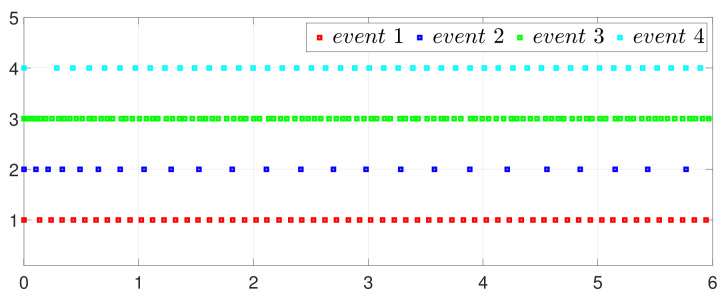
Event under one-to-all SET (2b).

**Figure 20:**
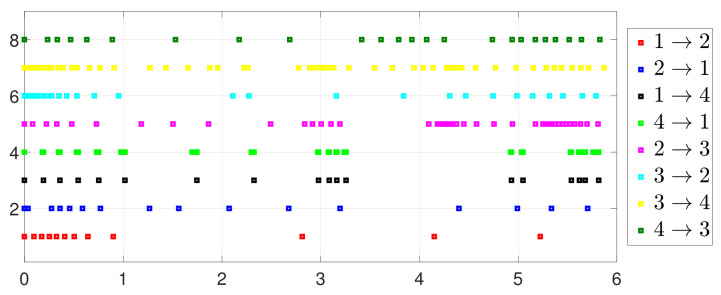
Event under one-to-one DET (5a).

**Figure 21:**
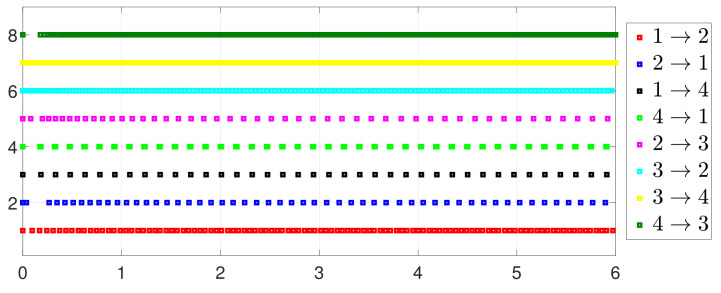
Event under one-to-one SET (5b).

By letting $\Gamma_{i}\left(t\right)=0$ and ${\tilde{\Gamma }}_{ij}\left(t\right)=0$ in (2) and (5), one has the one-to-all SET and one-to-one SET versions (2b) and (5b). Then, the one-to-all DET and SET strategies are compared in Figure 18 and Figure 19. Moreover, the one-to-one DET strategy and the corresponding SET strategy are compared in Figure 20 and Figure 21. Since $\Gamma_{i}\left(t\right)>0$ in (2) and ${\tilde{\Gamma }}_{ij}\left(t\right)>0$ in (5), the DET strategies are likely to have fewer triggering times, as compared with the SET strategies, which are also displayed in Figure 18, Figure 19, Figure 20 and Figure 21 and Table 3 and Table 4, which means that DET is beneficial for saving system resources with a slower update frequency.

## 6. Conclusions

In this paper, two novel DET strategies are combined to design distributed optimization algorithms to solve the RAP; they have fewer trigger times compared to SET strategies. Furthermore, the designed distributed optimization algorithms require only the state information of the agent itself and do not require information exchange with neighboring nodes, which saves on the communication energy of the system. Furthermore, the internal dynamic variables $\Gamma_{i}\left(t\right)$ and ${\tilde{\Gamma }}_{ij}\left(t\right)$ not only solve the RAP, but also play an important role in eliminating the Zeno behavior. In the future, we will combine DET strategies to study optimization problems with equality and inequality constraints under directed graphs.

## Figures and Tables

**Table 1:** Notation used in this paper.

Symbol	Description
R	A set of real numbers
$R^{n}$	An *n*-dimensional Euclidean space
∥·∥	The Euclidean norm or induced matrix 2-norm
N	{1,2,⋯,n}
$\mathrm{diag}\{\alpha_{1},\alpha_{2},\cdots ,\alpha_{n}\}$	A diagonal matrix with $\alpha_{i},\;i=1,2,\cdots ,n$
$1_{n}$	An n×1 column vector of all ones
$0_{n}$	An n×1 column vector of all zeros
$I_{n}$	An n×n identity matrix
A⊗B	The Kronecker product of matrices $A\in R^{m\times n}$ and $B\in R^{p\times q}$
$D^{+}f\left(x_{0}\right)$	The right-hand Dini derivative of *f* at $x_{0}$
∇f	The gradient of *f*

**Table 2:** Cost coefficients.

*i*	$\alpha_{i}$	$\beta_{i}$	$\gamma_{i}$
1	0.5	3	2
2	1.5	4	1
3	3	5	0.5
4	1	2	1.5

**Table 3:** One-to-all DET performance comparison with SET.

Event Triggering Strategy	Triggering Numbers for Agents			
	1	2	3	4
DET	12	24	45	22
SET	60	24	105	46

**Table 4:** One-to-one DET performance comparison with SET.

Event Triggering Strategy	Triggering Numbers for Agents							
	1→2	2→1	1→4	4→1	2→3	3→2	3→4	4→3
DET	12	16	18	211	44	28	55	24
SET	106	53	39	251	48	158	1038	1073

## Data Availability

Not applicable.

## Abbreviations

The following abbreviations are used in this manuscript:

MASs	Multi-agent systems
SET	Static event-triggered
DET	Dynamic event-triggered
RAP	Resource allocation problem
